# Structural Elucidation and Molecular Docking of a Novel Antibiotic Compound from Cyanobacterium *Nostoc* sp. MGL001

**DOI:** 10.3389/fmicb.2016.01899

**Published:** 2016-11-29

**Authors:** Niveshika, Ekta Verma, Arun K. Mishra, Angad K. Singh, Vinay K. Singh

**Affiliations:** ^1^Laboratory of Microbial Genetics, Department of Botany, Banaras Hindu UniversityVaranasi, India; ^2^Department of Chemistry, Banaras Hindu UniversityVaranasi, India; ^3^Centre for Bioinformatics, School of Biotechnology, Banaras Hindu UniversityVaranasi, India

**Keywords:** *Nostoc* sp. MGL001, novel bioactive compound, antibacterial agent, molecular docking, RNA fragments, OmpF porin protein

## Abstract

Cyanobacteria are rich source of array of bioactive compounds. The present study reports a novel antibacterial bioactive compound purified from cyanobacterium *Nostoc* sp. MGL001 using various chromatographic techniques *viz*. thin layer chromatography (TLC) and high performance liquid chromatography (HPLC). Further characterization was done using electrospray ionization mass spectroscopy (ESIMS) and nuclear magnetic resonance (NMR) and predicted structure of bioactive compound was 9-Ethyliminomethyl-12-(morpholin - 4 - ylmethoxy) -5, 8, 13, 16–tetraaza–hexacene - 2, 3 dicarboxylic acid (EMTAHDCA). Structure of EMTAHDCA clearly indicated that it is a novel compound that was not reported in literature or natural product database. The compound exhibited growth inhibiting effects mainly against the gram negative bacterial strains and produced maximum zone of inhibition at 150 μg/mL concentration. The compound was evaluated through *in silico* studies for its ability to bind 30S ribosomal fragment (PDB ID: 1YRJ, 1MWL, 1J7T, and 1LC4) and OmpF porin protein (4GCP, 4GCQ, and 4GCS) which are the common targets of various antibiotic drugs. Comparative molecular docking study revealed that EMTAHDCA has strong binding affinity for these selected targets in comparison to a number of most commonly used antibiotics. The ability of EMTAHDCA to bind the active sites on the proteins and 30S ribosomal fragments where the antibiotic drugs generally bind indicated that it is functionally similar to the commercially available drugs.

## Introduction

High proportion of drug resistance in bacterial pathogens indicated loss of efficacy of conventional antibiotics as only one third of the diseases could be cured by currently available drugs (Karchmer, 2004; Reynolds et al., 2004; Paterson, 2006). Thus, screening of new biologically active compounds are major thrust area at the present moment (Lahlou, 2013). To date, modern scientific advances in drug discovery could not enable the pace of newer drug development because of very little exploration of natural resources especially microbial metabolites (Cragg and Newman, 2013). Actinomycetes, fungi, unicellular bacteria along with cyanobacteria contributed about 45, 38, and 17%, respectively, in producing bioactive metabolites (Berdy, 2005; El-Elimat et al., 2012). Of these organisms, cyanobacteria are photoautotrophic in nature and can grow in presence of small amount of nutrients (Bullerjahn and Post, 2014; Dias et al., 2015). Due to these reasons, utilization of cyanobacteria in scientific studies will be a cost effective approach. Cyanobacteria constitute a rich source of unprecedented novel biologically active metabolites (Singh et al., 2005; Sivonen and Börner, 2008; Prasanna et al., 2010), such as peptides, macrolides, phenolic dilactones, polyketides, and alkaloids each of which originate from different pathway and show a broad spectrum of biological activities (Namikoshi and Rinehardt, 1996; Clardy and Walsh, 2004; Kim and Lee, 2006). Estimate proclaim regarding bioactive compounds in fresh water cyanobacteria shown to exhibit antimicrobial, antifungal, antiviral, antitumor, anticancer, and other pharmacological activities (Gul and Hamam, 2005; Mayer and Hamann, 2005; Singh et al., 2005). Extensive screening programme of cyanobacterial bioactive compounds for antibiotics, pharmaceutical and agricultural application has received considerable attention during the past few decades (Patterson et al., 1994; Khairy and El-Kassas, 2010; Kumar et al., 2010).

Among the cyanobacterial genera screened, *Nostoc* sp. are distributed throughout tropical and subtropical regions and proved as prodigious procedure of secondary metabolites. Genus *Nostoc* is highly diversified and reported from various terrestrial and aquatic habitats, and also able to form stable cyanobiont in various symbiosis (Dodds et al., 1995). *Nostoc* species attracted much attention as number of secondary metabolites were isolated, examined and found to have antiviral and antitumor properties (Dembitsky and Rezanka, 2005). Novel antimitotic compound namely Nostodione A has been reported from *Nostoc commune* (Kobayashi et al., 1994). *Nostoc commune* produced novel extracellular diterpenoid having antibacterial activity (Jaki et al., 1999). Potent antitumor agent and antifungal peptolides *viz*. Cryptophycins which is a cyclic depsipeptides isolated from *Nostoc* sp. showing excellent activity against broad spectrum of drug sensitive and drug resistant solid tumors, implanted in mice (Trimurtulu et al., 1994). An antiviral compound Cyanovirin-N has been isolated from *Nostoc ellipsosporum*. Freshwater *Nostoc spongiaeforme* produced Nostocine A exhibiting adverse effect on growth of microorganisms, algae, cultured plants and animal cell lines as well (Hirata et al., 2003). Boron containing metabolite, Borophycin isolated from marine strains *viz. Nostoc linckia* and *N spongiaeforme* var. *tenue*, and cryptophycin from *Nostoc* sp. ATCC 53789 and GSV 224 has been found to exhibit potent cytotoxicity against human tumor cell lines (Burja et al., 2001).

Therefore, in the present study cyanobacterium *Nostoc* sp. MGL001 isolated from fresh water body was used for the screening of antibacterial bioactive compound. Various chromatographic techniques like thin layer chromatography (TLC) and high performance liquid chromatography (HPLC) were performed for purification and purified compound were then subjected to electrospray ionization mass spectrometry (ESIMS) and nuclear magnetic resonance (NMR) spectroscopic analysis for identification and structure elucidation. Additionally, the design approaches mentioned above coupled with the *in silico* computational toolkit for optimizing interactions between ligand (bioactive compound) and receptor molecules. Here, 30S ribosomal fragment (1YRJ, 1MWL, 1J7T, and 1LC4) (Vicens and Westhof, 2001, 2002, 2003; Han et al., 2005) as well as OmpF porin protein (4GCP, 4GCQ and 4GCS) (Ziervogel and Roux, 2013) was selected as target receptors for molecular docking with ligand.

## Materials and methods

### Isolation and identification of cyanobacterium

The experimental organism cyanobacterium *Nostoc* sp. MGL001 was collected from local fresh water pond (Kardmeshwar pond) Chitaipur, Varanasi, India (25.2719° N, 82.9676° E). Pond has an area 5012 m^2^ with the mean depth approximately 10.3 m. This pond is present in vicinity of the adjacent temples and not connected to any river.

Sample was washed several times with sterile water and unialgal population of cyanobacterial strain was obtained by serially diluting the source inocula and subsequently streaking it on the solidified BG-11 agar medium. The purity of culture was routinely checked by streaking cyanobacterial culture on nutrient agar plates containing 0.5% of the glucose (w/v) incubated for 24 h. This process was repeated multiple times until pure micro-colonies were obtained. Further cyanobacterial culture was grown in 500 mL Erlenmeyer flasks containing BG11 medium (Rippka et al., 1979). The flasks were kept at 25 ± 2°C under white cool fluorescent light at an intensity of 95 μmol m^−2^ s^−1^, with a 14/10 h light/dark cycle. Identification of axenic cyanobacterial strain was based on the morphological features (Desikachary, 1959; Komarek, 2013) as well as 16S ribosomal gene amplification (Nübel et al., 1997). The light microscope (Dewinter) attached with a camera was used to study the morphology of filaments and cells. The cell dimensions were measured using software (Dewinter Biowizard 4.1).

To amplify the 16S ribosomal gene segment, DNA was extracted from the exponentially grown cyanobacterial culture growing on BG11 media using Himedia Bacterial DNA purification kit (MB505). Concentration of DNA was measured using Bio Spec Nano Spectrophotometer Life Science (Shimadzu Biotech). Eluted DNA was stored in −20°C. Amplification of 16S rDNA gene was performed using 16S rDNA bacteria specific primers 27F forward (5′-AGAGTTTGATCCTGGCTCAG-3′) and 1492R reverse (5′-TACGGTTACCTTGTTACGACTT-3′) (Weisburg et al., 1991). The PCR amplification (BioRad, DNA Engine, Peltier Thermal Cycler) of 16S rDNA was performed using 25 μl aliquots containing 20–50 μg DNA template, 0.4 μM of each primers, 1.5 μM MgCl_2_, 200 μM dNTPs, and 1 U/μl Taq Polymerase. Program followed for PCR amplification was: initial denaturation at 95°C for 3 min, 30 cycles of 30 s denaturation at 94°C, 40 s annealing at 55°C, and 50 s extension at 72°C and final extension at 72°C for 20 min (Singh et al., 2015). The amplified product was analyzed on a 1.2% agarose gel stained with ethidium bromide in 1X TBE buffer and then visualized under gel documentation system. The obtained bands were further cut down and eluted using Qiagen quick Gel Extraction kit. The eluted amplified products were finally sent to Sci Genome Cochin, Kerala, India for sequencing.

### Nucleotide sequence analysis and construction of phylogenetic tree

The partial 16S rDNA sequence obtained from DNA sequencing was then subjected to NCBI sequence database *viz*. nucleotide basic local alignment search tool (Blastn) (http://blast.ncbi.nlm.nih.gov/Blast.cgi) and aligned with the already existing gene sequences of different cyanobacterial species. Furthermore, the partial 16S rDNA sequence of the experimental organism was submitted to the NCBI database using the Sequin submission tool version 15.10. Evolutionary history of closely related sequence in a BLAST search was inferred by neighbor joining algorithm method for construction of phylogenetic tree (Saitou and Nei, 1987). Evolutionary analysis was performed using MEGA 6 software (Tamura et al., 2013).

### Preparation of crude extract

For the preparation of crude extract 40–45 days old cyanobacterial culture was used based on the method of Doan et al. (2000). Cyanobacterial cells (10 g fresh weight) were harvested by centrifugation at 10,000 rpm for 15 min (Remi, India) and then lyophilized (Christ-Alpha 1-2, Germany). Lyophilized cyanobacterial biomass (5 g) was extracted twice or thrice in 300 mL methanol (100%) by keeping it on shaker (150 rpm for 48 h) and centrifuged at 10,000 rpm (15 min). Supernatant was evaporated to dryness using rotary vacuum evaporator (Perfit, India) at 40°C and redissolved in 5 mL of 100% methanol for further use.

### Thin layer chromatography (TLC)

Dried crude extract of *Nostoc* sp. MGL001 was dissolved in methanol and purified by TLC (TLC silica gel 60, Merck, Darmstadt, Germany). In this process, carbon tetrachloride: methanol (9:1) was used as the mobile phase and silica was used as the stationary phase. The UV-illuminated orange bands on the TLC plate were designated as A, B, C, D, E, and F, which were dissolved separately in 100% methanol (1 mL). Further each elute was then subjected to TLC purification using ethyl acetate: n-hexane (1:1). Only the potent designated bands of the first step were subjected to the second step.

### Purification of the antibacterial compound produced by *Nostoc* sp. MGL001 by high performance liquid chromatography (HPLC) fractionation

Potent band elute after second step of TLC was dissolved in HPLC grade methanol. Separation was achieved using HPLC (Waters, USA) equipped photodiode array (PDA) detector and inline degasser. The reverse phase Nova pack C_18_ Spherisorb S10 ODS column (4.6 × 150 mm, 5 mm particle size) was used and temperature maintained at 40°C. Mobile phase consists of Milli-Q water (A) and acetonitrile (B), containing 0.05% trifluoroacetic acid. All solvents used in study were HPLC grade and filtered through a 0.22 μm membrane filter. For mobile phase, linear gradient programme was: 0 min 25% B, 35 min 70% B, 37 min 70% B, 38 min 25% B, 60 min 25% B (Lawton et al., 1994). Flow rate of 1 ml min^−1^ was maintained for sample run and separation was monitored at 200–300 nm (Harada et al., 1999) having 1.2 nm resolution. Empower2 software was used for instrumentation and data acquisition.

### Antibacterial assay

Pure fraction of bioactive compound was collected through HPLC and concentrated using rotary vacuum evaporator. Concentrated solid mass of pure bioactive compound were weigh. In order to perform antibacterial assay different concentrations of pure bioactive compound were prepared *viz*. 100, 150, 200, and 250 μg/mL in the volume of 1% methanol separately. Antibacterial activity was tested by agar disc diffusion method using 5 mm diameter filter paper discs. Agar was inoculated with a standardized quantity of the suspension of the test organisms. Known amount of pure bioactive compound were loaded on filter paper discs. Control discs received only the solvent (1% methanol). Discs were allowed to remain at room temperature until complete solvent evaporation and then placed on the seeded agar plates. The diameter of the inhibition zone (from periphery of disc to periphery of zone) was measured in mm after 24 h incubation at 37°C.

### ESI-MS and NMR studies

Electrospray ionization (ESI) equipped with time of flight mass spectrometer were recorded on Bruker daltonics AmaZon SL. Capillary voltage 3500 V in positive ion mode was set having 20 μL/min flow rate. The ^1^H, H-H COSY, ^13^C{^1^H}, DEPT-135, and DEPT-90 NMR spectra were recorded on a Bruker AVANCE III HD-500 MHz multinuclear FTNMR spectrometer at 25°C.

### Optimization of isolated bioactive compound and docking study

For visualization of isolated compound Discovery Studio 3.0 tool was used (Gao and Huang, 2011).

### Selection of target structures

The small ribosomal subunit (30S) fragment and protein structures were selected for interaction with isolated bioactive compound (ligand). The 3-D crystal structure of the target *viz*. the RNA fragments (1YRJ, 1MWL, 1J7T, and 1LC4) (Vicens and Westhof, 2001, 2002, 2003; Han et al., 2005) and OmpF porin structures (4GCP, 4GCQ, and 4GCS) (Ziervogel and Roux, 2013) were retrieved from the protein databank (PDB) (www.rcsb.org/pdb) and further used for interaction calculation using YASARA software (Krieger and Vriend, 2014). Active site of the targeted protein was predicted using MetaPocket 2.0 (http://projects.biotec.tu-dresden.de/metapocket/) (Huang, 2009; Zhang et al., 2011).

### Receptor preparation and docking

The complexes of RNA fragments and porin protein with heteroatom, water molecules and ligands were taken for receptor preparation. The heteroatom, water molecules and ligands were removed using Discovery Studio 3.1 before docking. The Docking calculation was performed for RNA fragments and porin protein with ligand (bioactive compound). The target protein was set in YASARA to run macro file (dock_run.mcr). The YASARA structure provides Autodock and VINA tools to dock ligands with proteins at the touch of a button (Morris et al., 1998; Trott and Olson, 2010).

## Results

### Isolation and identification

**Figure 1:**
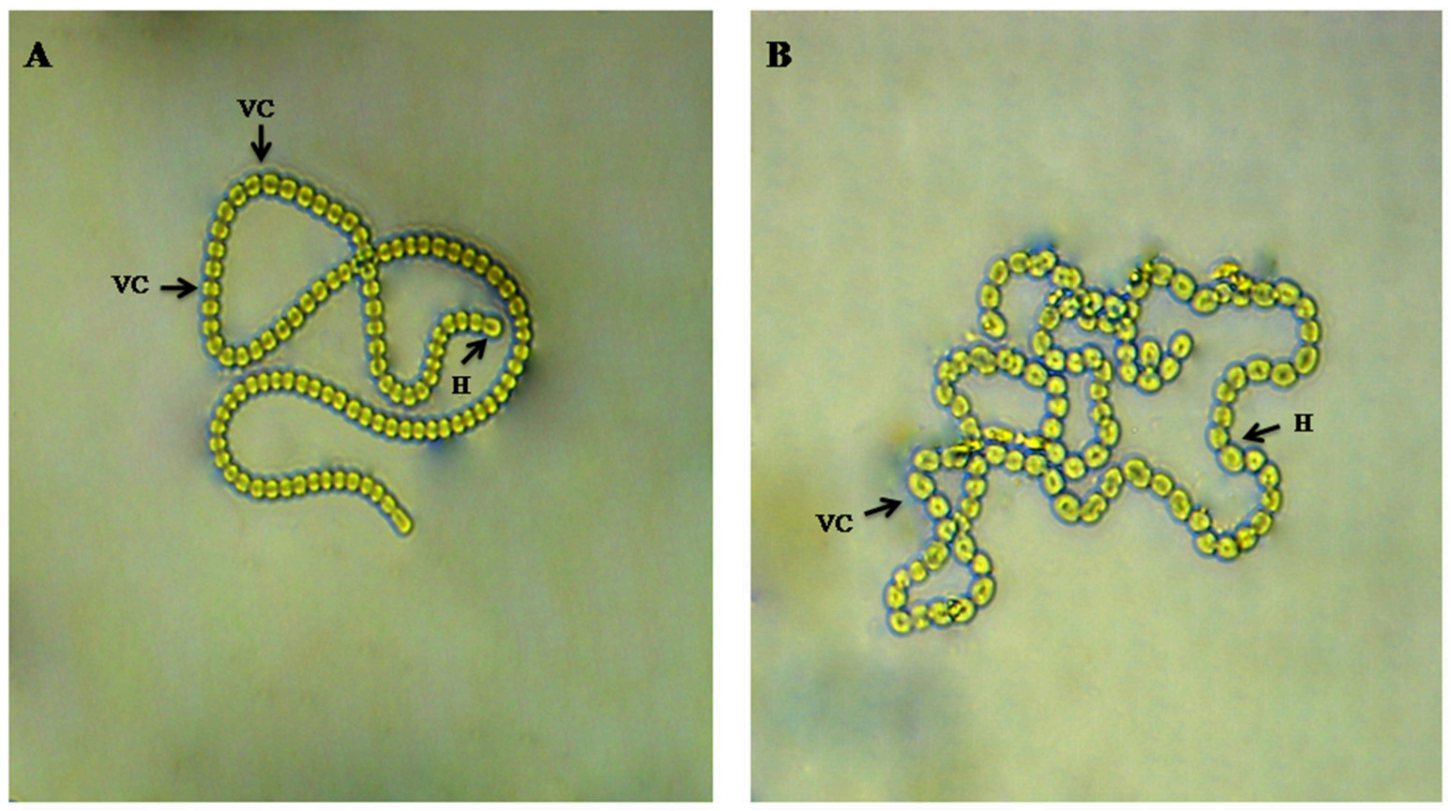
**Microphotographs of ***Nostoc*** sp. MGL001. (A)** and **(B)** showing filament with vegetative cell (VC) and heterocysts (H).

**Figure 2:**
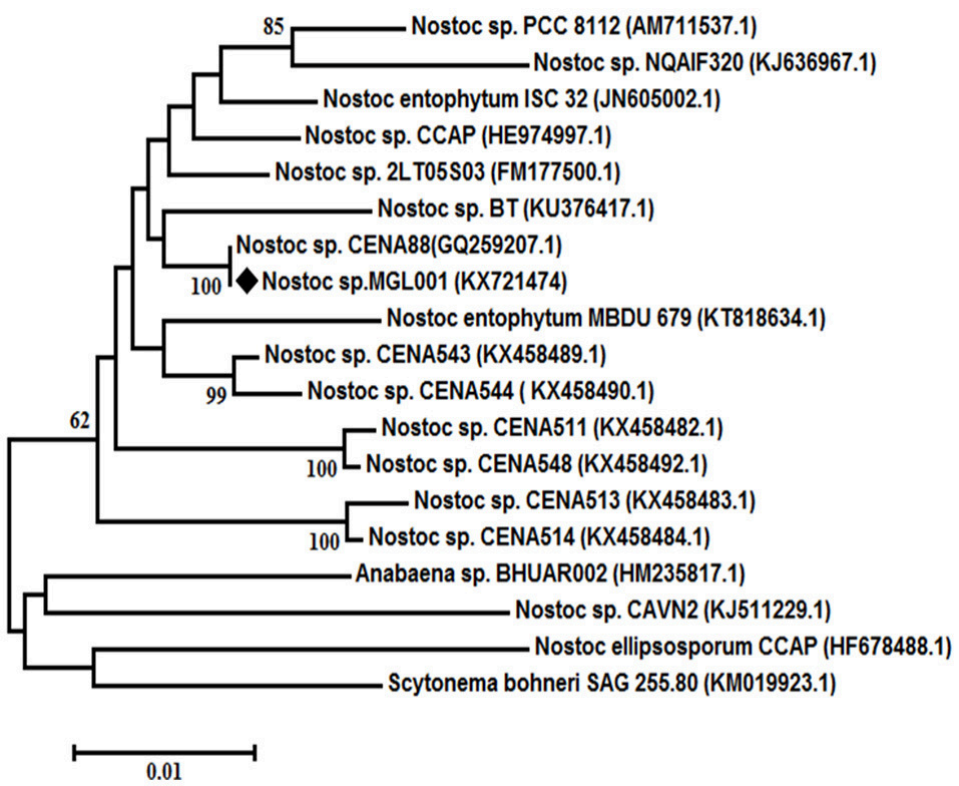
**Neighbor-joining tree of ***Nostoc*** sp MGL001 with reference strains using 16S rDNA gene sequences**. Bootstrap values (%) are based on 1000 replicates and shown at the branch point with more than 50% bootstrap values. ♦ Representing the cyanobacteriaum *Nostoc* sp. MGL001 used in the present study.

The morphological study of the cyanobacterium *Nostoc* sp. MGL001 showed that it contains squarish to cylindrical vegetative cells (Figure 1). *Nostoc* sp. MGL001 (GenBank, accession no. KX721474) and other close homologs for the cyanobacterium can be found from the alignment view tree (Figure 2).

### TLC and HPLC analysis

**Figure 3:**
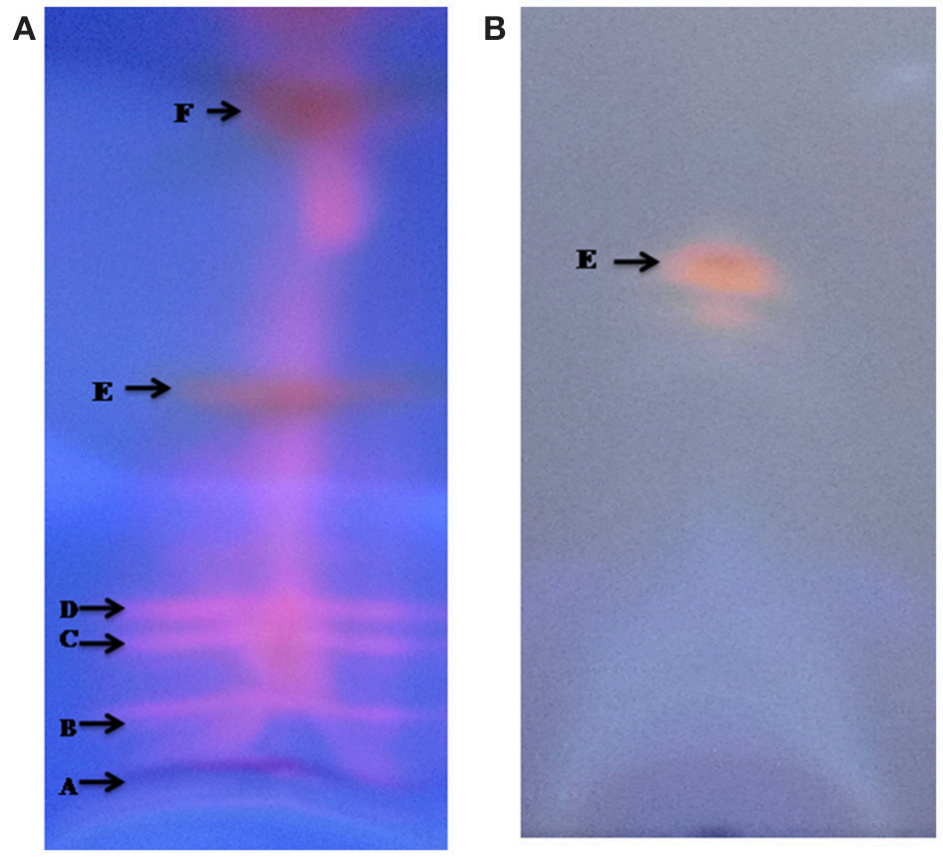
**TLC pattern of methanolic crude extract from ***Nostoc*** sp. MGL001 (A)** using carbon tetrachloride and methanol (9:1) as mobile solvent. **(B)** TLC pattern of 2nd TLC spot using ethyl acetate and hexane (1:1) as mobile solvent.

**Figure 4:**
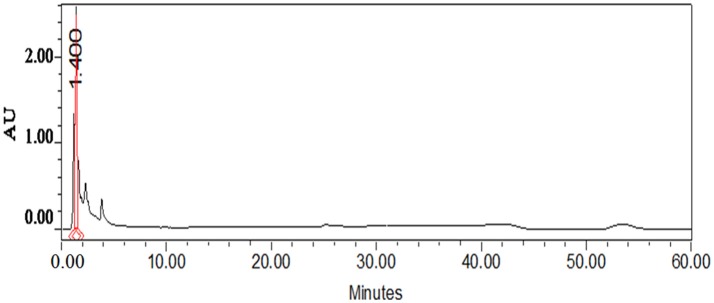
**HPLC chromatogram of the spot “E” fraction of the ***Nostoc*** sp. MGL001 crude extract obtained from TLC**.

Thin layer chromatography (TLC) of the crude extract of cyanobacterium produced five bands A, B, C, D, E, and F (Figure 3). Only band E showed potent antibacterial property. Further band E was purified by HPLC, the individual peak with retention time (Rt) of 1.40 min (Figure 4) were collected.

### Antibacterial bioassay

This purified compound shows extensive antimicrobial activity against various gram negative bacterial strains chosen, such as *Escherichia coli* (KX758560), *Proteus vulgaris* (KX758561), and *Pseudomonas aeruginosa* (KX758562). Maximum zone of inhibition at a concentration of 150 μg/mL of pure compound isolated from *Nostoc* sp. MGL001 was observed *viz*. 11.15 ± 0.117, 10.17 ± 0.235, and 9.16 ± 0.211 mm against *Escherichia coli, Proteus vulgaris* and *Pseudomonas aeruginosa*, respectively (Table 1). Upon further increasing the concentration upto 250 μg/mL, no further increase in the size of zone was observed.

**Table 1:** **Antibacterial activity of the EMTAHDCA compound against gram negative bacteria**.

**Test organism**	**Inhibition zone (mm) at different concentrations of EMTAHDCA (μg/mL)**			
	**100**	**150**	**200**	**250**
*E. coli*	4.78±0.324	**11.15 ± 0.117**	11.13±0.123	11.15±0.133
*P. vulgaris*	5.22±0.221	**10.17 ± 0.235**	10.16±0.353	10.16±0.271
*P. aeruginosa*	4.11±0.356	**9.16 ± 0.211**	9.15±0.233	9.15±0.279

### ESI and NMR

**Figure 5:**
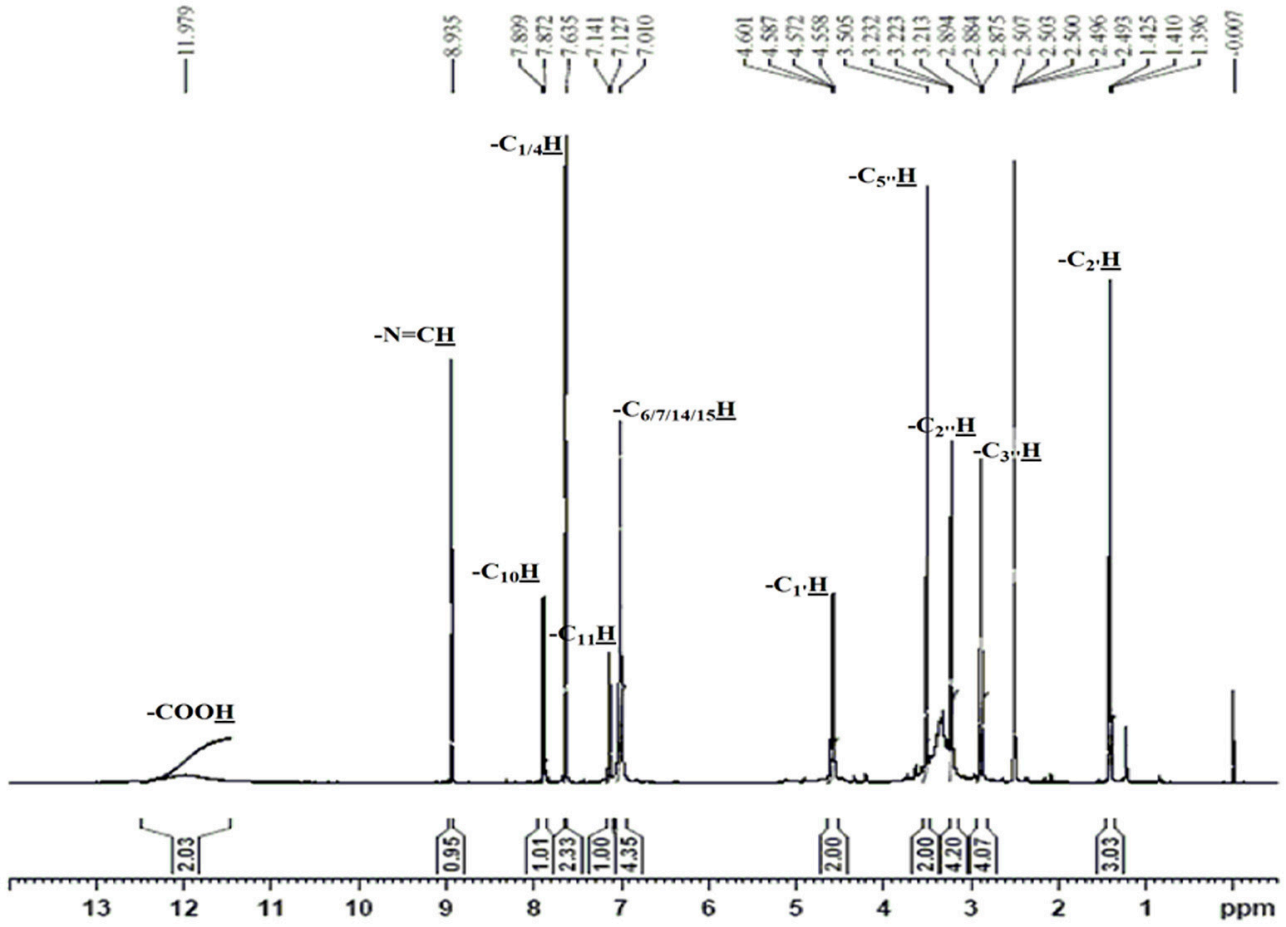
**^**1**^H NMR spectrum of the bioactive compound (spot “E” eluate) derived from ***Nostoc*** sp. MGL001**.

**Figure 6:**
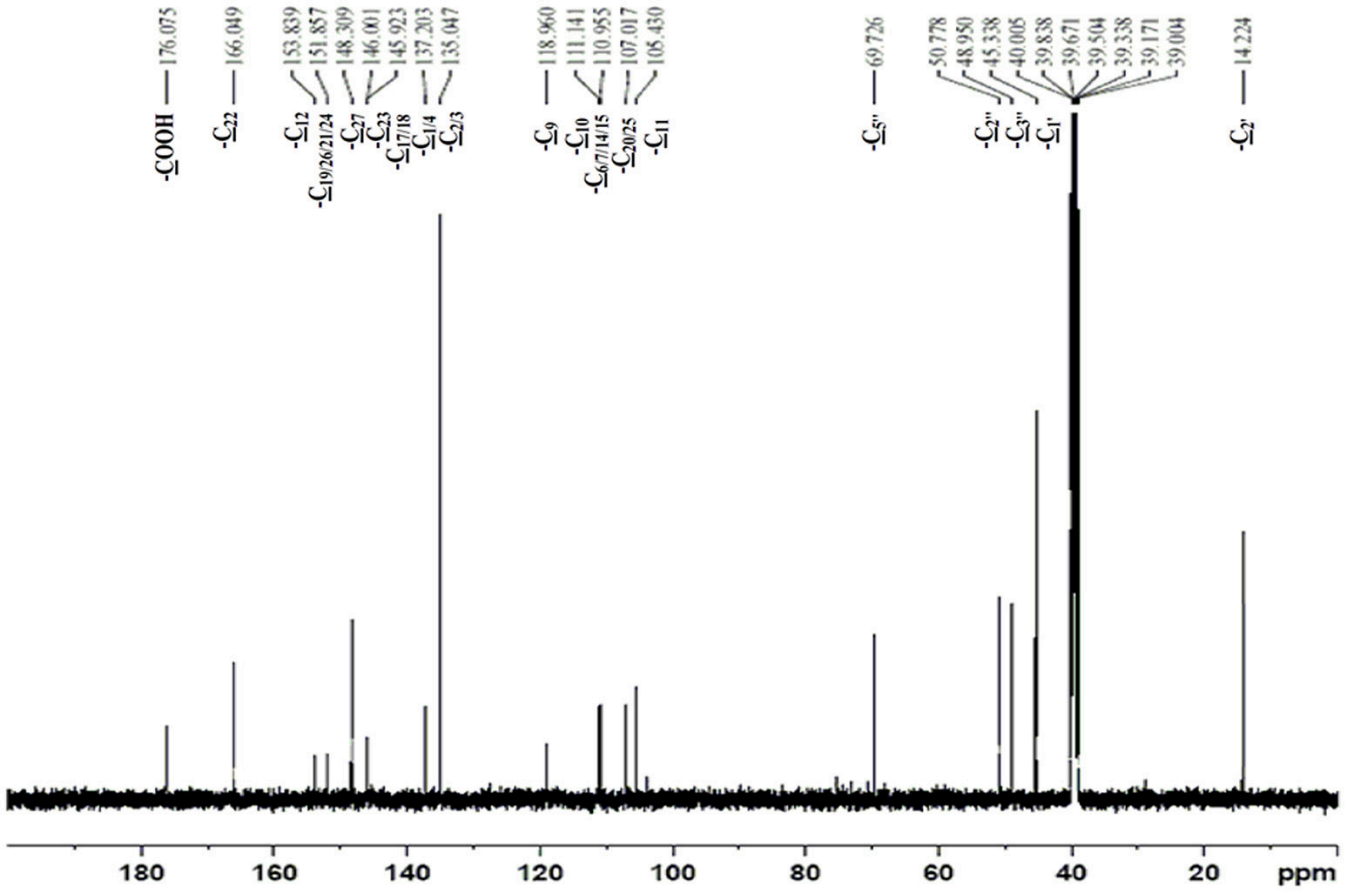
**^**13**^C NMR spectrum of the bioactive compound (spot “E” eluate) derived from ***Nostoc*** sp. MGL001**.

**Figure 7:**
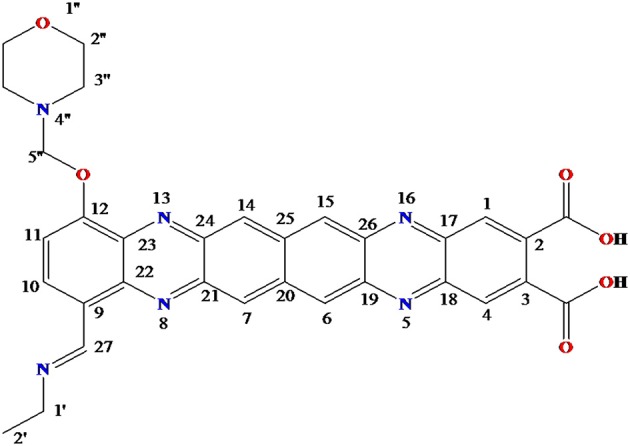
**Proposed structure of bioactive compound was 9-Ethyliminomethyl-12-(morpholin - 4 - ylmethoxy) - 5, 8, 13, 16 - tetraaza - hexacene - 2, 3 dicarboxylicacid (EMTAHDCA)**.

The obtained whitish powder compound was used for the analysis through ESI and NMR. The ^1^H NMR (500.30 MHz;) δ ppm w.r.t. DMSO-d_6_(2.500) (Figure 5) and ^13^C{^1^H} NMR (125.80 MHz) δ ppm w.r.t. DMSO-d_6_(39.51) (Figure 6) in DMSO-*d*_*6*_ at 25°C spectral data are given in Table 2. Therefore, on the basis of NMR, proposed structure of bioactive compound was 9-Ethyliminomethyl-12-(morpholin - 4 - ylmethoxy) -5, 8, 13, 16–tetraaza–hexacene - 2, 3- dicarboxylic acid (EMTAHDCA) (Figure 7). High resolution ESI-MS was operated in positive ion mode revealed an m/z at 591.02 (M+H^+^) corresponding to the molecular formula C_32_H_26_N_6_O_6_ (Molecular weight-590.59) assigned in full agreement with NMR spectral data.

**Table 2:** **NMR Spectral data of EMTAHDCA**.

**S. No**.	**^1^H/^13^C{^1^H} (Multiplicity)**	**No. of proton**	**Peak position (δ)**	**Coupling constant ^3^*J_*H-H*_*(Hz)**
1	-COOH (s)	02	11.979	–
2	-N = CH (s)	01	8.935	–
3	-C_10_H (d)	01	7.899–7.872	13.50
4	-C_1/4_H(s)	02	7.635	–
5	-C_11_H(d)	01	7.141–7.127	7.0
6	-C_6/7/14/15_H (s)	04	7.010	–
7	-C_1′_H (q)	02	4.601–4.558	7.5/7.5
8	-C_5″_H (s)	02	3.505	–
9	-C_2″_H (t)	04	3.232–3.213	5.0
10	-C_3″_H (t)	04	2.894–2.875	5.0
11	-C_2′_H (t)	03	1.425–1.396	7.0
12	-COOH	–	176.08	–
13	-C_22_	–	166.05	–
14	-C_12_	–	153.84	–
15	-C_19/26/21/24_	–	151.86	–
16	-C_27_	–	148.31	–
17	-C_23_	–	146.00	–
18	-C_17/18_	–	145.92	–
19	-C_1/4_	–	137.20	–
20	-C_2/3_	–	135.05	–
21	-C_9_	–	118.96	–
22	-C_10_	–	111.14	–
23	-C_6/7/14/15_	–	110.96	–
24	-C_20/25_	–	107.02	–
25	-C_11_	–	105.43	–
26	-C_5″_	–	69.73	–
27	-C_2″_	–	50.78	–
28	-C_3″_	–	48.95	–
29	-C_1′_	–	45.34	–
30	-C_2′_	–	14.22	–

*s, singlet; q, quartet; d, doublet; t, triplet*.

### Molecular docking

**Figure 8:**
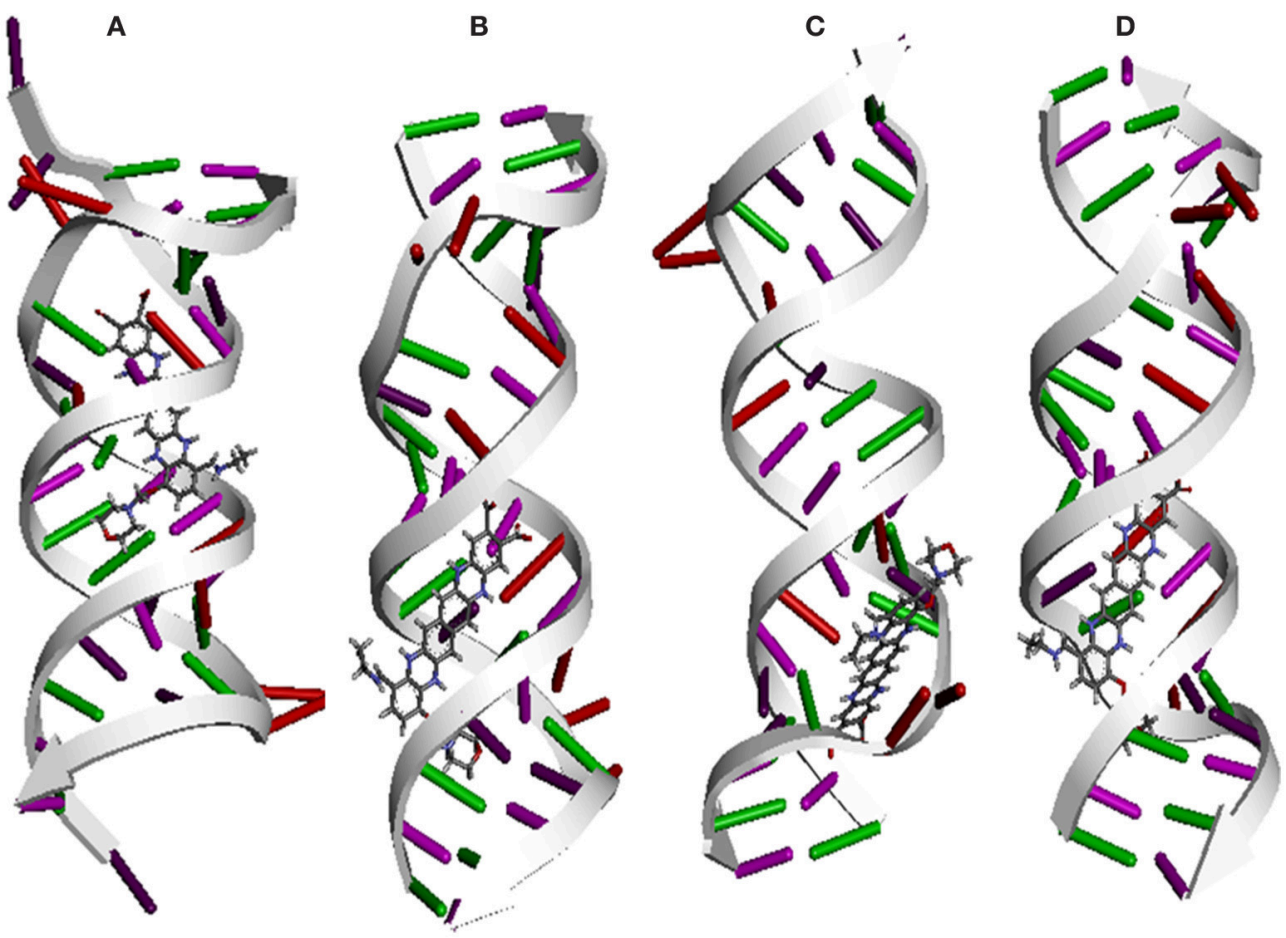
*****In silico*** interaction between selected ligand (EMTAHDCA) and target RNA fragment (A)** 1YRJ **(B)** 1MWL, **(C)** 1J7T, **(D)** 1LC4.

**Figure 9:**
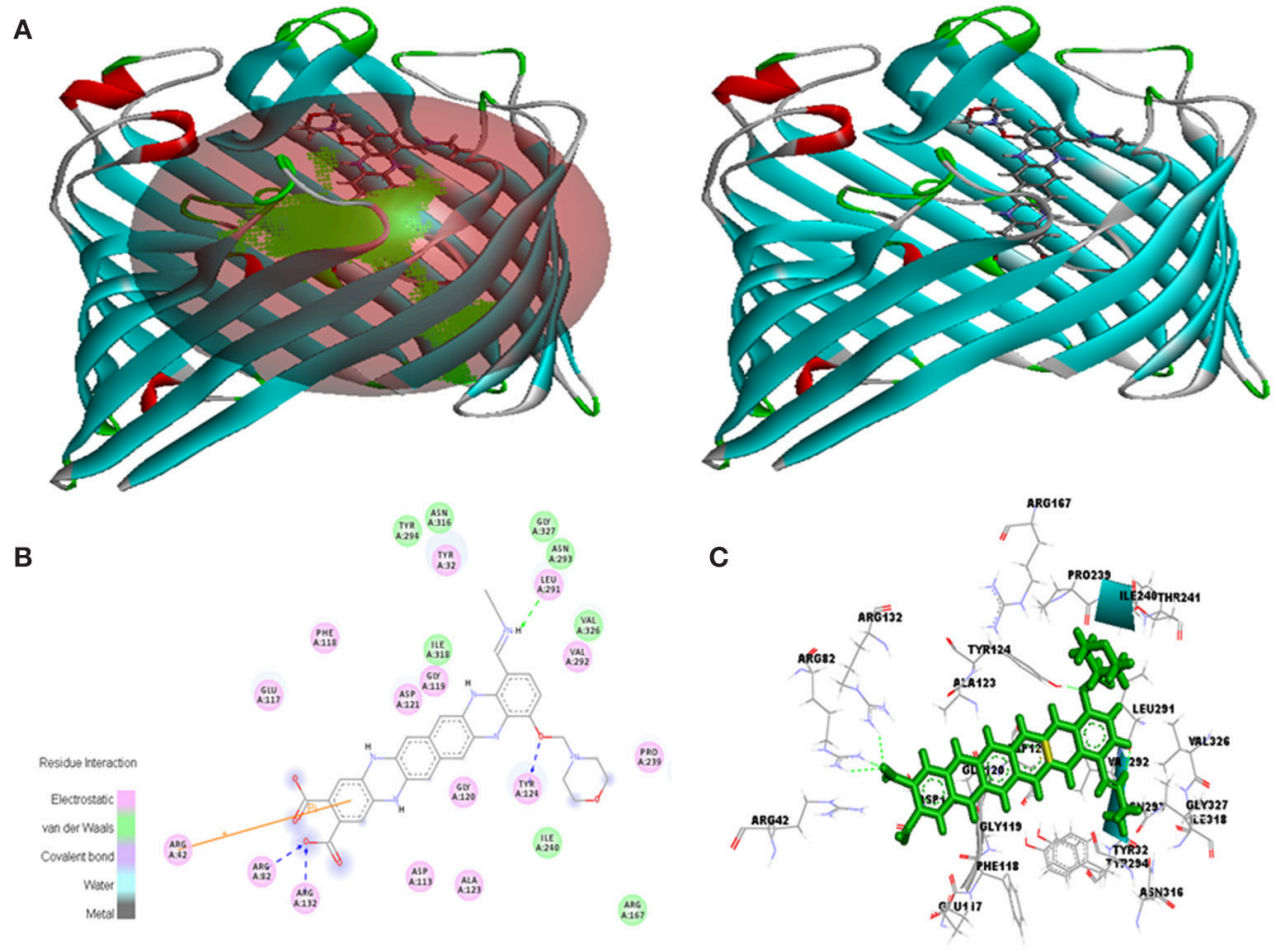
*****In silico*** interaction between selected ligand (EMTAHDCA) and target OmpF porin protein (4GCP). (A)** Poses of docked complexes, green color in sphere indicates prominent active site where the ligand interacted, **(B)** 2D level interaction, **(C)** 3D level interaction.

**Figure 10:**
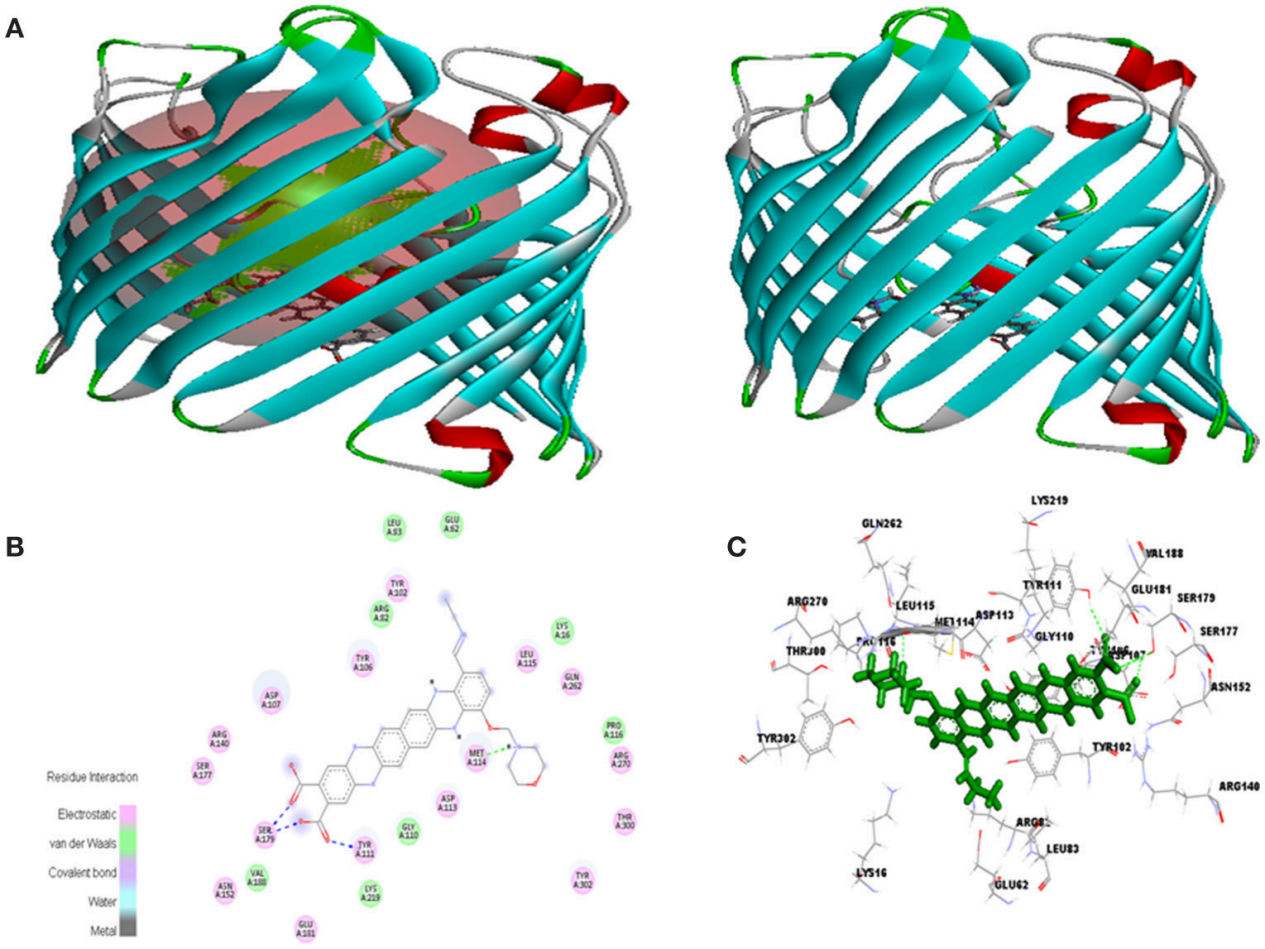
*****In silico*** interaction between selected ligand (EMTAHDCA) and target OmpF porin protein (4GCQ). (A)** Poses of docked complexes, green color in sphere indicates prominent active site where the ligand interacted, **(B)** 2D level interaction, **(C)** 3D level interaction.

**Figure 11:**
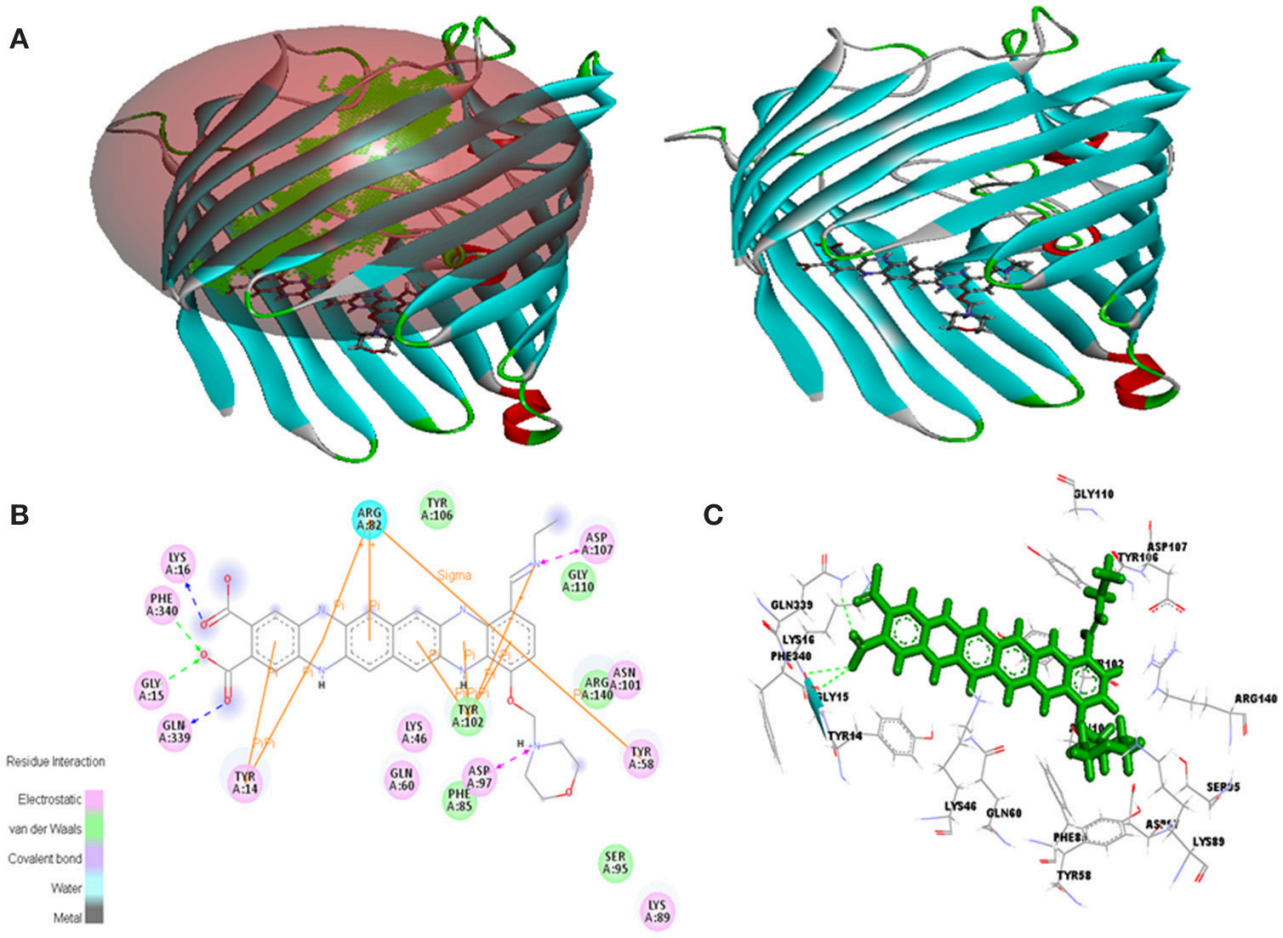
*****In silico*** interaction between selected ligand (EMTAHDCA) and target OmpF porin protein (4GCS). (A)** Poses of docked complexes, green color in sphere indicates prominent active site where the ligand interacted, **(B)** 2D level interaction, **(C)** 3D level interaction.

Molecular docking was successfully performed between selected ligand (EMTAHDCA) and target receptor RNA fragments (PDB ID: 1YRJ, 1MWL, 1J7T, and 1LC4) (Figure 8) and OmpF porin protein (4GCP, 4GCQ, and 4GCS) (Figures 9, 10, 11) using YASARA software. The active site of OmpF was determined using Metapocket and results are represented in Table 3. Summary of molecular docking results of selected ligand with drug target RNA fragment and OmpF porin protein are represented in Table 4. The binding energy was found to be 000011.1450, 000010.0550, 000009.8620, and 000009.9690 for 1YRJ, 1MWL, 1J7T, and 1LC4 RNA fragments, respectively. Dissociation constant [pM] was found to be 00000000006770.2808, 00000000042617.4336, 00000000059028.0117, and 00000000059028.0117 for 1YRJ, 1MWL, 1J7T, and 1LC4 RNA fragments, respectively (Table 4). Contacting receptor residues were identified through YASARA software and found that G^A5^, U^A6^, A^A8^, A^A10^, A^B33^, G^B39^, and U^A15^ were common active site residues between reported 1YRJ complex with Apramycin and selected ligand.

**Table 3:** **Prominent active site residues identification of selected Omp porin protein models (4GCP, 4GCQ, and 4GCS)**.

**S.No**.	**Target**	**PDB ID**	**Common active site 1 residues**
1.	OmpF porin protein	4GCP	Arg^42^, Arg^82^, Tyr^32^, Leu^291^, Val^292^, Asn^293^, Tyr^294^, Asn^316^, Ile^318^, Val^326^, Asp^121^, Gly^327^, Tyr^124^, Pro^239^, Ile^240^, Thr^241^, Phe^118^, Gly^119^, Gly^120^, Arg^167^, Glu^117^, Ala^123^, Asp^113^, Arg^132^
2.		4GCQ	Lys^16^, Arg^82^, Pro^116^, Tyr^302^, Thr^300^, Leu^115^, Tyr^102^, Asp^113^, Met^114^, Arg^270^, Tyr^106^, Gln^262^, Glu^62^, Leu^83^, Gly^110^, Asp^107^, Tyr^111^, Arg^140^, Lys^219^, Glu^181^, Ser^179^, Val^188^, Asn^152^, Ser^177^
3.		4GCS	Lys^89^, Ser^95^, Arg^140^, Tyr^58^, Asp^97^, Asp^107^, Phe^85^, Asn^101^, Gln^60^, Tyr^106^, Lys^46^, Tyr^102^, Gly^110^, Tyr^14^, Lys^16^, Phe^340^, Gln^339^, Gly^15^

**Table 4:** **Summary of molecular docking results of selected ligand with drug target RNA fragment and OmpF porin protein**.

**S.No**.	**Ligand**	**Target**	**PDB ID**	**Binding energy [kcal/mol]**	**Dissociation constant [pM]**	**Contacting receptor residues**	**Reported active site residues**	**Common**
1.	EMTAHDCA	RNA Fragment	1YRJ	000011.1450	00000000006770.2808	G^A5^, U^A6^, C^A7^, A^A8^, A^A10^, C^A11^, C^A12^, G^A13^, U^A15^, A^B33^, C^B34^, C^B35^, G^B37^, U^B38^, G^B39^	C^A4^, G^A5^, U^A6^, A^A8^, A^A10^, U^A15^, C^A4^, G^A5^, U^A6^, A^A8^, A^A10^, U^*A*15^, G^A16^, A^A17^, A^A18^, G^A19^, U^A20^, U^B25^, A^B31^, C^B32^, A^B33^, G^B39^, A^B40^, A^B41^	G^A5^, U^A6^, A^B33^, G^B39^, A^A8^, A^A10^, U^A1^, U^A15^, A^B33^, G^B39^
2.	EMTAHDCA	RNA Fragment	1MWL	000010.0550	00000000042617.4336	C^A10^, C^A11^, G^A12^, G^A13^, U^A14^, C^A20^, G^B26^, U^B27^, C^B28^, C^B30^, A^B31^, C^B32^, C^B33^, G^B34^	A^B33^, G^B39^, G^A4^, U^A5^, A^A6^, C^A7^, C^A7^, C^A8^, G^A15^, A^A16^, A^A17^, G^A18^, U^A19^, C^B25^, G^B26^, U^B27^, C^B28^, A^B29^, C^B30^, G^B37^, A^B38^, A^B39^, G^B40^, U^B41^	G^B26^, U^B27^, C^B28^, C^B30^
3.	EMTAHDCA	RNA Fragment	1J7T	000009.8620	00000000059028.0117	C^A10^, C^A11^, G^A12^, G^A13^, U^A14^, G^A18^, U^A19^, C^A20^, C^B25^, G^B26^, U^B27^, C^B28^, A^B29^, C^B30^, A^B31^, C^B32^, C^B33^, G^B34^	G^A4^, U^A5^, C^A6^, A^A7^, U^A17^, G^A15^, A^A16^, A^A17^, G^A18^, U^A19^, G^B26^, U^B27^, C^B28^, A^B29^, C^B30^, U^B36^, G^B37^, A^B38^, A^B39^, G^B40^	G^A18^, U^A19^
4.	EMTAHDCA	RNA Fragment	1LC4	000009.9690	00000000049274.9414	U^A15^, G^A16^, A^A17^, A^A18^, G^A19^, U^A20^, C^A21^, C^B27^, G^B28^, U^B29^, C^B30^, A^B31^, C^B32^, A^B33^, C^B34^	C^A4^, G^A5^, C^A7^, G^A16^, A^A18^, G^A19^, U^A20^, C^B27^, G^B28^, C^B30^, A^B31^, G^B39^, A^B41^, G^B42^, U^B43^	G^A16^, A^A18^, G^A19^, U^A20^, C^B27^, G^B28^, C^B30^, A^B31^
5.	EMTAHDCA	OmpF Porin protein	4GCP	000009.8890	00000000056398.4141	Tyr^32^, Arg^42^, Arg^82^, Asp^113^, Glu^117^, Phe^118^, Gly^119^, Gly^120^, Asp^121^, Ala^123^, Tyr^124^, Arg^132^, Arg^167^, Pro^239^, Ile^240^, Thr^241^, Leu^291^, Val^292^, Asn^293^, Tyr^294^, Asn^316^, Ile^318^, Val^326^, Gly^327^	Ser^31^, Tyr32, Gly^33^, Phe^118^, Gly^119^, Gly^120^, Asp^121^, Tyr^124^, Lys^243^, Leu^291^, Val^292^, Asn^316^, Val^326^, Gly^327^	Tyr^32^, Phe^118^, Gly^119^, Gly^120^, Asp^121^, Tyr^124^, Leu^291^, Val^292^, Asn^316^, Val^326^, Gly^327^
6.	EMTAHDCA	OmpF porin protein	4GCQ	000009.9500	00000000050880.7266	Lys^16^, Glu^62^, Arg^82^, Leu^83^, Tyr^102^, Tyr^106^, Asp^107^, Gly^110^, Tyr^111^, Asp^113^, Met^114^, Leu^115^, Pro^116^, Glu^181^, Val^188^, Lys^219^, Gln^262^, Arg^270^, Thr^300^, Tyr^302^	Tyr^22^, Tyr^32^, Gly^33^, Arg^42^, Arg^82^, Glu^117^, Phe^118^, Gly^119^, Gly^120^, Asp121 Arg^132^, Val ^292^	Arg^82^
7.	EMTAHDCA	OmpF porin protein	4GCS	000008.6880	00000000428165.4063	Tyr^14^, Gly^15^, Lys^16^, Lys^46^, Tyr^58^, Gln^60^, Phe^85^, Lys^89^, Ser^95^, Asp^97^, Asn^101^, Tyr^102^, Tyr^106^, Asp^107^, Gly^110^, Arg^140^, Gln^339^, Phe^340^	Thr^165^, Arg^168^, Ser^248^	

In case of 1MWL RNA fragment bases C^A10^, C^A11^, G^A12^, G^A13^, U^A14^, C ^A20^, G^B26^, U^B27^, C^B28^, C^B30^, A^B31^, C^B32^, C^B33^, and G^B34^ were involved in interaction between ligand and 1MWL RNA fragment, in which G^B26^, U^B27^, C^B28^, and C^B30^ bases were common active site residues between reported Geneticin antibiotic complex (1MWL) and ligand.

The ligand and 1J7T RNA fragment interacted with the following binding residues C^A10^, C^A11^, G^A12^, G^A13^, U^A14^, G^A18^, U^A19^, C^A20^, C^B25^, G^B26^, U^B27^, C^B28^, A^B29^, C^B30^, A^B31^, C^B32^, C^B33^, and G^B34^ in which G^A18^, U^A19^, G^B26^, U^B27^, C^B28^, A^B29^, and C^B30^ bases were common between reported paromomycin antibiotic complex (1J7T) and ligand (Table 4).

Active site residues *viz*. U^A15^, G^A16^, A^A17^, A^A18^, G^A19^, U^A20^, C^A21^, C^B27^, G^B28^, U^B29^, C^B30^, A^B31^, C^B32^, A^B33^, and C^B34^ were involved in interaction with 1LC4 RNA fragment and ligand in which G^A16^, A^A18^, G^A19^, U^A20^, C^B27^, G^B28^, C^B30^, and A^B31^ bases were common binding residues between reported tobramycin antibiotic complex (1LC4) and ligand.

In case of 4GCP porin protein docking results, site 1 residues Tyr^32^, Arg^42^, Arg^82^, Asp^113^, Glu^117^, Phe^118^, Gly^119^, Gly^120^, Asp^121^, Ala^123^, Tyr^124^, Arg^132^, Arg^167^, Pro^239^, Ile^240^, Thr^241^, Leu^291^, Val^292^, Asn^293^, Tyr^294^, Asn^316^, Ile^318^, Val^326^, and Gly^327^ were involved in interaction with good positive energies and dissociation constant. The site 1 is the major prominent site for interaction of any compound. The residues of site 1 (Tyr^32^, Phe^118^, Gly^119^, Gly^120^, Asp^121^, Tyr^124^, Leu^291^, Val^292^, Asn^316^, and Val^326^) are common to the reported active site known against ampicillin antibiotics (Figure 9, Table 4). The 2D and 3D view of interacted residues also mentioned in Figure 9. In residue interaction electrostatic bond, van der waals, covalent bond and hydrogen bond were involved.

Molecular docking between 4GCQ porin protein and ligand indicates that active site 1 residues Lys^16^, Glu^62^, Arg^82^, Leu^83^, Tyr^102^, Tyr^106^, Asp^107^, Gly^110^, Tyr^111^, Asp^113^, Met^114^, Leu^115^, Pro^116^, Glu^181^, Val^188^, Lys^219^, Gln^262^, Arg^270^, Thr^300^, and Tyr^302^ were involved in interaction, whereas only Arg^82^ are common to the reported active site known against Carbenicillin antibiotics (Figure 10, Table 4). The electrostatic bond, van der waals, covalent bond and hydrogen bond were involved in residues interaction could be clearly visible in 2D and 3D view in Figure 10.

Active site 1 residues involved in interaction were Phe^85^, Ser^95^, Asn^101^, Tyr^102^, Tyr^106^, Gly^110^, and Arg^140^ in case of 4GCS porin protein and ligand docking through van der waals bonding, Residues like Tyr^14^, Lys^16^, Gly^15^, Phe^34^, Gly^15^, Gln^339^, Asp^97^, Lys^46^, Lys^89^, Tyr^58^, Asn^101^, and Asp^107^ involved in electrostatic bonding whereas Arg^82^ involved in hydrogen bond interaction (Figure 11, Table 4).

## Discussion

Cyanobacteria are known to produce a wide variety of biologically active compounds. To the best of our knowledge, this type of new bioactive compound EMTAHDCA is first time reported from fresh water cyanobacterium *Nostoc* sp. MGL001. In order to isolate EMTAHDCA various chromatographic techniques were tested but TLC was found to be most useful tool to achieve the separation of complex mixtures of organic molecules. Competition between solute and the mobile phase is responsible for the separation of compounds through TLC (Kumar et al., 2013). In order to separate unknown bioactive compounds, different gradients of solvents were tested and ultimately carbon tetrachloride: methanol in 9:1 ratio was found to be best for separation point of view. Same solvent composition was also used by Srivastava et al. (2015) to separate bioactive compound from fresh water cyanobacteria *Geitlerinema* sp. CCC728 and *Arthrospira* sp. CCC729.

Liquid chromatography is one of the most efficient and powerful separation methods for the preparative purification and isolation of biological substances (Nikitas and Pappa-Louisi, 2009). A number of different chromatographic methods were considered for purification of bioactive compounds but reason behind selection of HPLC technique was its reproducibility, sensitivity, high resolution, and absence of extensive sample preparation or derivatization (Quilliam, 2003; Snyder and Dolan, 2006). Water is the weakest eluent for reverse phase HPLC so; its eluent strength is then modified by adding methanol or acetonitrile which are less polar but miscible solvents. After testing methanol and acetonitrile based systems acetonitrile was selected as mobile phase because it gave better peak shapes, stable baselines and sharper resolution as compared to methanol.

**Figure 12:**
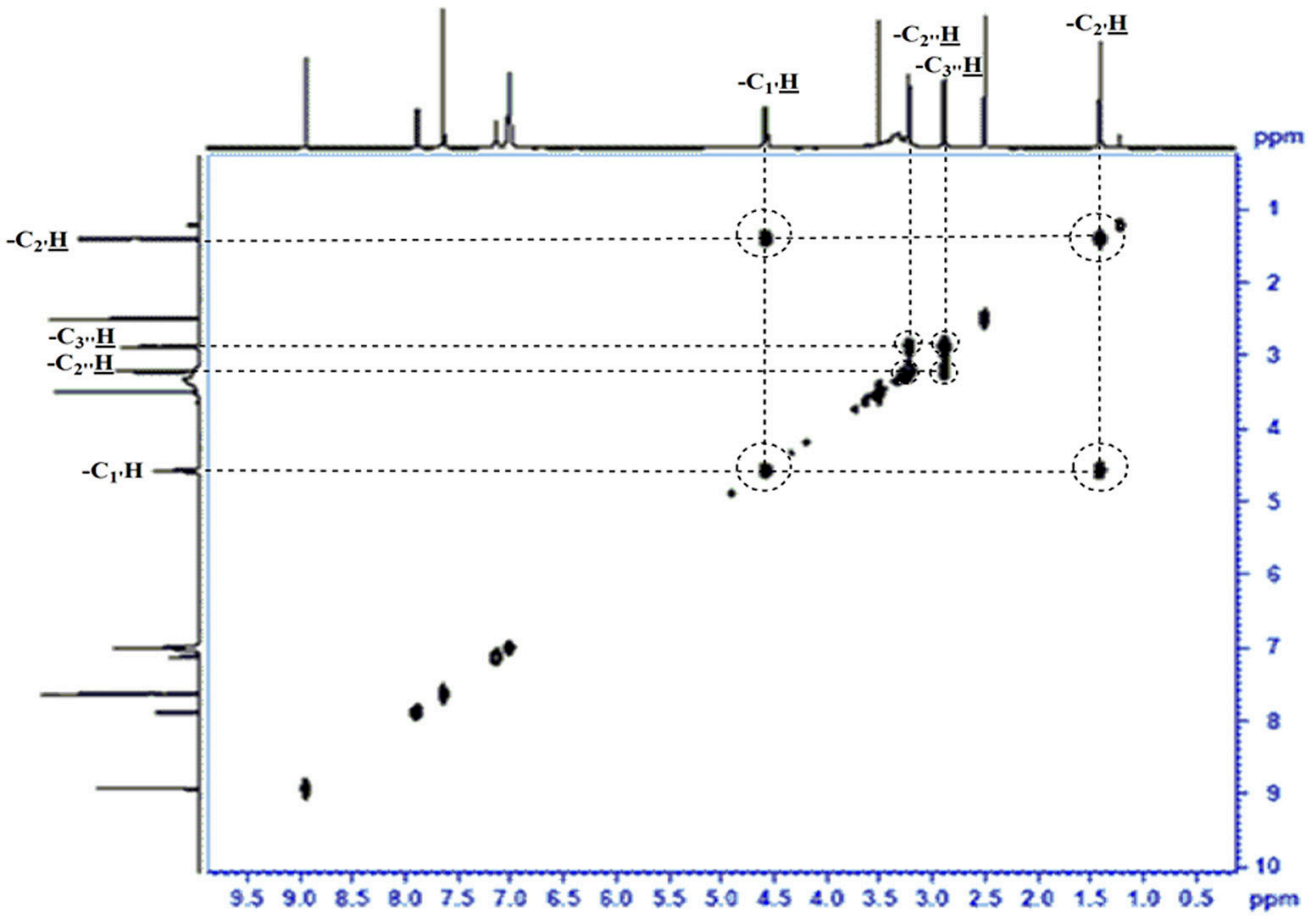
**H-H COSY NMR spectrum of EMTAHDCA**.

**Figure 13:**
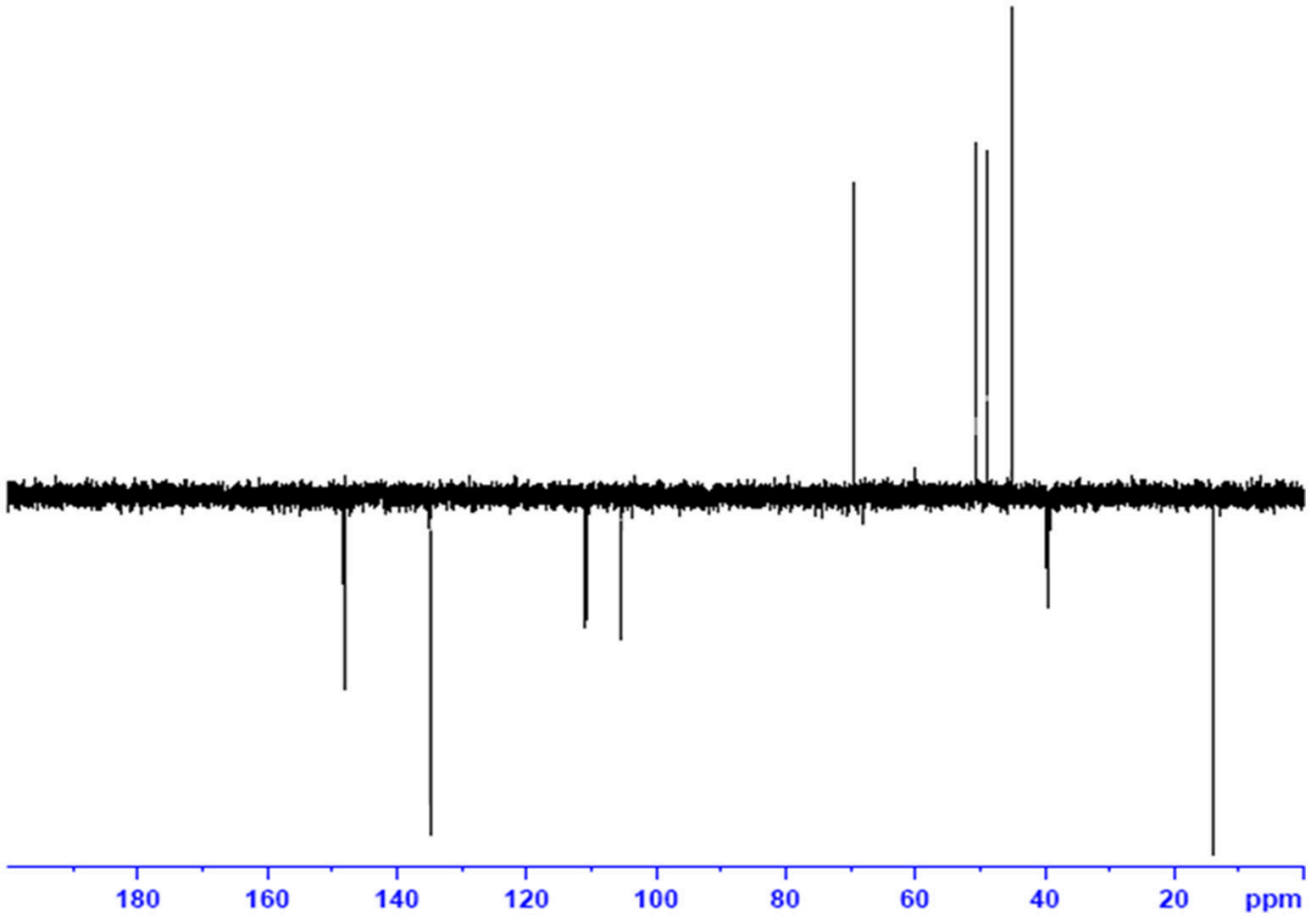
**DEPT-135 NMR spectrum of EMTAHDCA**.

**Figure 14:**
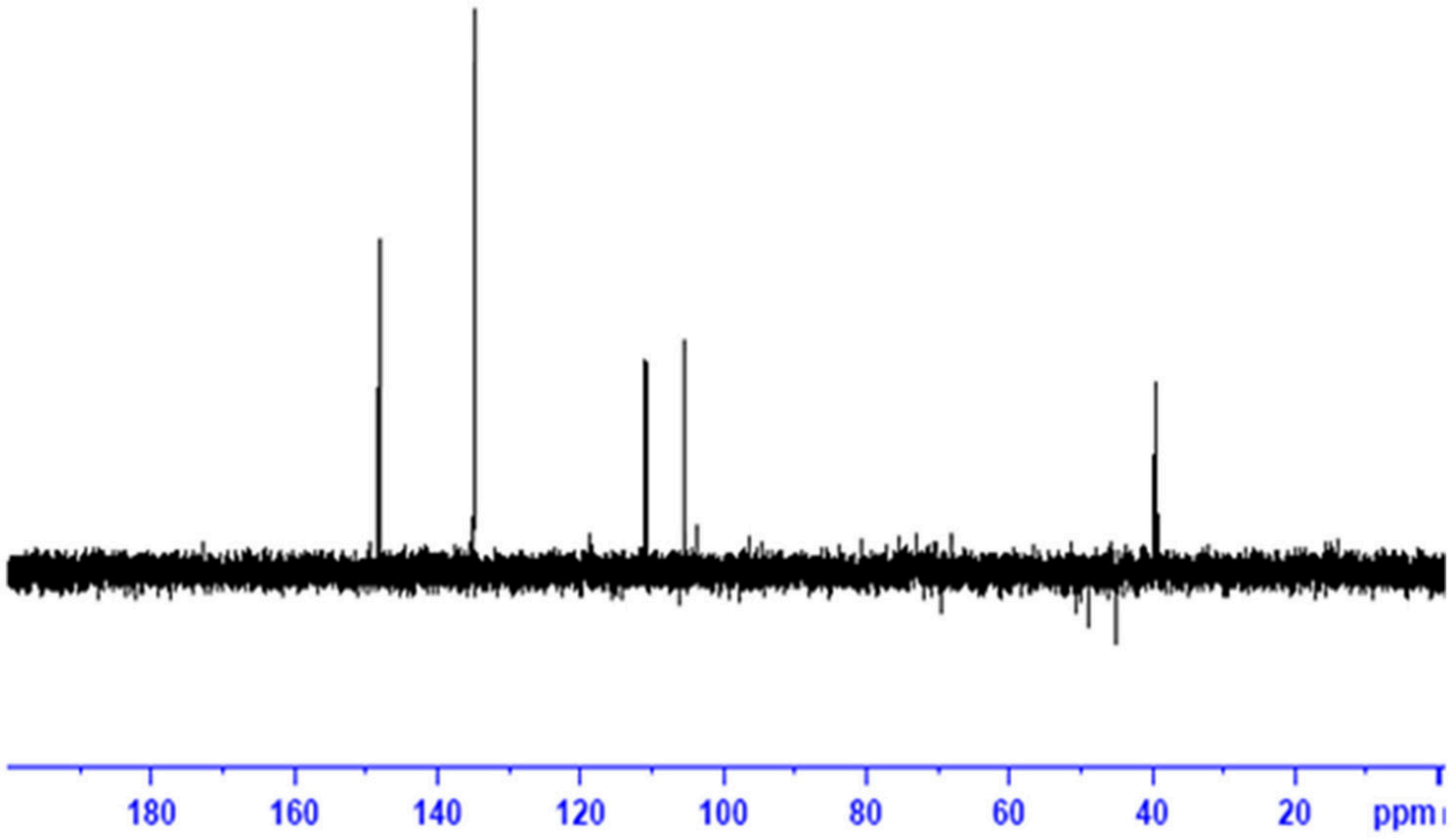
**DEPT-90 NMR spectrum of EMTAHDCA**.

The use of ESI-MS systems when coupled with a HPLC-system increases their selectivity and benefits from both techniques (Vishwakarma and Rai, 2013). For the structure elucidation of newly isolated compounds NMR methods have been used as it is very much capable of elucidating intact biomaterials nondestructively without any preceding derivatization (Willmann et al., 2011). Due to the simplicity of sample preparation and ease of interpretation of characteristic signals, NMR was favored as natural products are closely related and difficult to separate. The ^1^H and ^13^C{^1^H} NMR spectral data of EMTAHDCA has already been presented and interpreted in result section. The proton NMR spectrum of the EMTAHDCA (Figure 5), as per the structure, is expected to show a broad peak at δ 11.979 (due to presence two acidic proton of –COOH groups), two doublet [at δ 7.899–7.872 (due to presence of one aromatic proton, such as C_10_H) and at δ 7.141–7.127 (due to presence of one aromatic proton, such as C_11_H)], four singlet [at δ 8.935 (due to presence of one proton –N = CH group), δ 7.635 (due to presence of two aromatic protons, such as C_1_H and C_4_H), δ 7.010 (due to presence of four aromatic protons, such as C_6_H, C_7_H, C_14_H, and C_15_H) and at δ 3.505 (due to presence of two protons of methylene group, such as C_5″_H)], one quartet at δ 4.601–4.558 (due to presence of two protons of methylene group, such as C_1′_H) and three triplets [at δ 3.232–3.213(due to presence of two protons of methylene group, such as C_2″_H), δ 2.894–2.875(due to presence of two proton of methylene group, such as C_3″_H) and at δ 1.425–1.396 (due to presence of three protons of methyl group, such as C_2′_H)]. The splitting pattern of different type of protons of aliphatic region also confirmed by H-H COSY spectrum (Figure 12), in this spectrum the methylene group protons (C_1′_H) coupled with the methyl group protons (C_2′_H) and other methylene group protons (C_2′_H) coupled with another methylene group protons (C_3′_H). In the ^13^C{^1^H} NMR spectrum (Figure 6), 19 carbon signals were observed, including carbonyl carbon (δ 176.08), one imine carbon (δ 148.31), four aromatic carbons (δ 137.20, 111.14, 110.96, and 105.43), eight different type quaternary aromatic carbons (δ 166.05, 153.84, 151.86, 146.00, 145.92, 135.05, 118.96, and 107.02), four different type methylene carbons (δ 69.73, 50.78, 48.95, and 45.34), and one methyl carbon (δ 14.22). The different type of carbon interpreted with help of DEPT-90 and DEPT-135 NMR spectral technique, in DEPT-135 spectrum (Figure 13), carbon with one hydrogen (four type aromatic carbons and one imine carbon) and three hydrogens (one methyl carbon) shows positive peak, whereas carbon has two hydrogens (four different type of methylene carbons) shows negative peak and in DEPT-90 spectrum (Figure 14), carbon with one hydrogen (four type aromatic carbons and one imine carbon) shows only positive peak. The spectrum consists of all the expected signals as shown in spectral data of NMR section. The predicted structure of EMTAHDCA using ESI-MS and NMR spectra were verified by subjecting predicted structure on Pubchem (https://pubchem.ncbi.nlm.nih.gov/) and PubMed NCBI https://www.ncbi.nlm.nih.gov/pubmed) and by comparing experimental data with published literature on bioactive compounds of cyanobacteria but structure of EMTAHDCA clearly indicated that it is a novel compound that was not reported earlier in any literature or natural product database.

The ESI-MS and NMR spectra were verified by comparing experimental data with published literature on bioactive compounds but structure of EMTAHDCA clearly indicated that it is a novel compound that was not reported in literature or natural product database.

Also EMTAHDCA identified as an antibacterial agent through *in vitro* and *in silico* approaches. The present work based on *in silico* preclinical evaluation of novel isolated compound EMTAHDCA to proceed ahead for further drug trials. Structure elucidation of bioactive compound EMTAHDCA (Ethyliminomethyl-12-(morpholin - 4 - ylmethoxy) -5, 8, 13, 16 - tetraaza - hexacene - 2, 3 dicarboxylic acid) was done through NMR already mentioned in results section. This compound contain morpholin moiety that is rare in nature. The 2-hydroxymorpholine moiety was also observed in bacilosarcins A isolated from marine-derived bacterium *Bacillus subtilis* TP-B0611 showed growth inhibition against barnyard millet (Azumi et al., 2008). Morpholin is commonly used in organic synthesis. It acts as a building block in the preparation of the anticancer agent gefitinib (Iressa), the antibiotic linezolid and the analgesic dextromoramide (Fung et al., 2001; McKillop et al., 2005). Presence of morpholin moiety in the bioactive compound EMTAHDCA is likely to be responsible for its antibiotic activity. Pure bioactive compound EMTAHDCA (150 μg/mL) possessed antibacterial activity against gram negative bacterial strains *viz. E. coli, P. vulgaris*, and *P. aeruginosa*. These strains were tested in laboratory and found multi drug resistant (MDR) to erythromycin, imipenem, ciprofloxacin and vancomycin (data not shown). Bacterial adaptation to antibiotics generated serious medical problem (Chalasani et al., 2015). This finding gives an idea that EMTAHDCA compound could be used as an alternative antibiotic against such resistant bacterial strains. In order to prove its antibiotic potential *in silico* technique was performed to know the interaction of selected bioactive compound EMTAHDCA with small ribosomal subunit (30S) (PDBID: 1YRJ, 1MWL, 1J7T, and 1LC4) fragment and OmpF porin protein (PDBID: 4GCP, 4GCQ, and 4GCS).

Protein synthesis is a fundamental process performed by ribosome. More than half of the total number of clinically used antibiotics exerts their antibiotic effects on the bacterial ribosomes by binding to several sites of 30 and 50S subunits ultimately blocking protein synthesis (Franceschi and Duffy, 2006). Both the 30S and the 50S ribosomal subunits provide functionally relevant active site pockets considered as ribofunctional loci where antibiotics do act (Wilson, 2014). The ribosomes are highly conserved organelles having precise conformational variation which facilitates drug selectivity for clinical use (Hermann, 2005).

The outer membrane (OM), unique to gram negative bacteria acts as selective barrier by providing an extra protective layer against a harsh environment. OM provides passage for nonspecific charged and zwitterionic nutrient molecules (Delcour, 2009). Three major general diffusion porins found in *E.coli* are OmpF, OmpC, and PhoE. In several reports, loss of OmpF, and OmpC porins are linked to antibiotic resistance, especially for *Escherichia coli* and *Salmonella typhimurium* (Nikaido, 2003). Therefore, a better understanding of how modification of membrane permeability triggers bacterial resistance to antibiotics is necessary for the development of new antibiotic therapy strategies.

Molecular docking calculation between EMTAHDCA and target receptor molecule (RNA fragment) showed good binding affinity with best positive energies for selected compound EMTAHDCA. More positive energies indicates stronger binding, and negative energies mean no binding (Krieger and Vriend, 2014). Bases involved in interaction between ligand and ribosomal fragment resides at the prominent active site of ribosomal fragment. Similar type of interaction was observed in the case of EMTAHDCA docking with OmpF porin protein. The crystal structures of OmpF in complex with ampicillin, carbenicillin and ertapenem (4GCP, 4GCQ, and 4GCS) have been already reported. Residues involved in interaction between selected ligand and OmpF porin proteins was found in prominent active site (site 1) predicted by Metapocket. Therefore, with the help of emerging molecular docking tools, we could able to prove that isolated compound EMTAHDCA have ability to work as an antibiotic agent and also recognize their precise mode of action against targeted host. This study suggested that selected compound EMTAHDCA could better act in comparison with reported antibiotics and able to serve as clinical candidate.

## Conclusion

Novel bioactive compound EMTAHDCA isolated from *Nostoc* sp. MGL001 has antibacterial activity against multi drug resistant bacterial strains *viz*. *E coli, P vulgaris*, and *P aeruginosa* through *in vitro* and *in silico* studies. Therefore, it is worth mentioning that EMTAHDCA could serve as potential antibiotic drug.

## Author contributions

Niveshika and AM designed the experiments. Niveshika performed the experiments. Niveshika, EV, AM, AS, and VS analyzed the data. Niveshika and EV wrote the manuscript and AM critically reviewed the paper.

### Conflict of interest statement

The authors declare that the research was conducted in the absence of any commercial or financial relationships that could be construed as a potential conflict of interest.
